# An updated methodology to review developing-country vaccine manufacturer viability

**DOI:** 10.1016/j.vaccine.2017.04.087

**Published:** 2017-07-05

**Authors:** Nicholas Luter, Ritu Kumar, Dai Hozumi, Tina Lorenson, Shannon Larsen, Bhavya Gowda, Amie Batson

**Affiliations:** a PATH, PO Box 900922, Seattle, WA 98109, USA; b Management Sciences for Health, 4301 North Fairfax Drive, Suite 400, Arlington, VA 22203, USA; c Bill & Melinda Gates Foundation, PO Box 23350, Seattle, WA 98102, USA

**Keywords:** Vaccine production, Developing-country, Manufacturer viability, cGMP, current Good Manufacturing Practice, DCVM, developing-country vaccine manufacturer, EPI, Expanded Programme on Immunization, MNC, multinational corporation, NRA, national regulatory authority, PAHO, Pan American Health Organization, TRIPS, Agreement on Trade-Related Aspects of Intellectual Property, UNICEF, United Nations Children’s Fund, WHO, World Health Organization

## Abstract

In 1997, Milstien, Batson, and Meaney published “A Systematic Method for Evaluating the Potential Viability of Local Vaccine Producers.” The paper identified characteristics of successful vaccine manufacturers and developed a viability framework to evaluate their performance. This paper revisits the original study after two decades to determine the ability of the framework to predict manufacturer success. By reconstructing much of the original dataset and conducting in-depth interviews, the authors developed informed views on the continued viability of manufacturers in low- and middle-income country markets. Considering the marked changes in the market and technology landscape since 1997, the authors find the viability framework to be predictive and a useful lens through which to evaluate manufacturer success or failure. Of particular interest is how incumbent and potentially new developing-country vaccine manufacturers enter and sustain production in competitive international markets and how they integrate (or fail to integrate) new technology into the production process. Ultimately, most manufacturers will need to meet global quality standards to be viable. As governments and donors consider investments in vaccine producers, the updated viability factors will be a useful tool in evaluating the prospects of manufacturers over the mid to long term. The paper emphasizes that while up-front investments are important, other critical factors—including investments in a national regulatory authority, manufacturer independence, and ability to adapt and adopt new technology—are necessary to ensure viability.

## Highlights

• 8 factors predict the viability of vaccine manufacturers in developing countries.
• These factors have evolved as the vaccine landscape has changed over 2 decades.
• A new analysis updates a framework first published in 1997 to assess viability.
• The updated framework is useful for assessing investments in vaccine manufacturers.

## 1 Introduction

In 1997, Milstien, Batson, and Meaney analyzed the characteristics of vaccine manufacturers in developing countries and proposed seven critical factors to predict their long-term viability as suppliers [1]. Milstien et al. utilized the seven factors as a lens through which to recommend interventions such as strategic investments and increased political advocacy to address identified shortcomings in vaccine production facilities and operations. Since then, the framework and the viability factors have been used to assess vaccine manufacturers and shape global vaccine strategies.

Most developing-country vaccine manufacturers (DCVMs) in 1997 were state owned. As governments prioritized immunization and vaccines in the 1980s, local manufacturing seemed a natural step toward vaccine self-sufficiency. Milstien et al.’s working definition of viability, which was developed within the 1990s context of self-sufficiency, reflects this focus: “the ability of governments to provide for a stable sustainable supply of high-quality vaccines to meet national demand, for current and for future vaccines” [1]. Today, DCVMs have evolved into a blend of public, parastatal, and private-sector manufacturers, supplying vaccines domestically, to other countries, and to international procurers, particularly the United Nations Children’s Fund (UNICEF) and the revolving fund of the Pan American Health Organization (PAHO). Given the shift from not only meeting national needs to also competing in international markets, we broaden the definition of viability to “the long-term ability of a vaccine producer to reliably provide adequate quantities of high-quality vaccines at an affordable, and economically viable price to meet demand*.*”

The widely held belief that life-saving vaccines should be sold at low, affordable prices to government and international procurement agencies places unique pressures on vaccine manufacturers, particularly given the difficulty and technical complexity of vaccine manufacturing compared with the production of other pharmaceutical products [2]. DCVMs are further challenged by changes in global market dynamics, increased sophistication of technological requirements, and the need for heightened regulatory rigor [3]. Furthermore, some manufacturers struggle with significant operational, quality, and managerial challenges.

Vaccine manufacturer viability continues to be important because immunization remains one of the most cost-effective health interventions to prevent deaths and illness from infectious diseases and saves millions of dollars of health care and other costs to society [4], [5], [6]. Using the framework set out by Milstien et al., we update the viability factors based on changes in vaccine markets, technological requirements, and regulatory standards since 1997. We then analyze the performance of manufacturers included in the original study according to their probable viability in 1997, updating the results of a seminal paper.

## 2 Methods

Through a literature search, we identified technological, regulatory, economic, and other developments that have affected DCVMs over the past 20 years and supplemented this information with data gathered during a series of qualitative interviews with experts. Table 1 summarizes key search terms and respondent profiles. With available resources that informed the primary dataset of Milstien et al., we assessed how the manufacturers in the original study fared over the 20-year time period. Using insights from this analysis, we confirmed and updated the viability factors, adapting the criteria to today’s environment.

**Table 1:** Profiles of respondents in expert interviews and selected terms for literature search.

Informant type	Number	Description
Technical experts	5	Experts included technical assistance providers to vaccine manufacturers, procurement agencies or funders, governments, and regulatory authorities. Areas of expertise included technical transfers, production, Good Manufacturing Practices, and business strategy
Procurement and technical assistance agencies	2	Manage pooled procurement and quality assurance on behalf of large donors and governments
Developing-country vaccine manufacturers	21	Manufacturers that supply to both national and international Expanded Programme on Immunization markets
Selected search terms for the literature search	Developing country vaccine manufacturers/Manufacturing, Emerging market vaccine manufacturers/Manufacturing, History of vaccine production/Manufacturing in developing countries, History of vaccine production/Manufacturing in emerging markets, Vaccine production/Manufacturing in: Africa/Asia/India/South America/Eastern Europe, Vaccine producer/Manufacturer viability, Vaccine, Producer/Manufacturer sustainability, Developing country vaccine markets, History of regulation of vaccines, WHO regulation of vaccines, History of Gavi, Good Manufacturing Practice (GMP), World Health Organization (WHO) vaccine prequalification process	

## 3 Results

### 3.1 The evolving vaccine market 1997–2016

The dramatic growth in demand for traditional and new vaccines resulted in increased emphasis on ensuring a “healthy” vaccine market, defined as a market with adequate supply, reliable quality, and appropriate prices to meet global and national demands for new and existing vaccines [7]. The vaccine market grew from $3 billion to $41 billion from the mid-1990s to 2016, at the same time that regulatory, investment, and competitive pressures created new challenges for DCVMs [8], [9], [10]. The key drivers of change for the vaccine landscape and DCVMs over the past 20 years are as follows.

1. *Development and introduction of new vaccines:* Technological advances have led to the development of new vaccines over the past two decades, including rotavirus, pneumococcal conjugate, meningococcal conjugate, and human papillomavirus vaccines. Simultaneously, pressure to reduce the number of injections per child and the complexity of the Expanded Programme on Immunization (EPI) schedule have fueled the development and increased adoption of multivalent vaccines such as pentavalent vaccine (DTwP-HepB-Hib) and measles, mumps, and rubella combination vaccines. The number of vaccine antigens recommended by the World Health Organization (WHO) for inclusion in the EPI schedule continues to rise, from 6 in 1974, to 8 in 1997, to between 12 and 15 today, depending on the country. In addition, WHO recommends 11 other antigens for high-risk areas or populations [11], [12].
2. *Increasing regulation:* As the vaccine market has evolved, so has the emphasis on high-quality production and safety of vaccines. Stringent current Good Manufacturing Practice (cGMP) standards, WHO prequalification requirements, and tighter oversight of and by national regulatory authorities (NRAs) require companies and countries to continually invest in equipment and facilities modernization and staff training to comply with quality and safety standards. The more robust standards increase the cost of vaccine production and largely define which markets manufacturers can enter. In addition, more rigorous enforcement of intellectual property rules with the Agreement on Trade-Related Aspects of Intellectual Property (TRIPS), together with the harmonization of patent laws globally, creates a more challenging environment for DCVMs to access new vaccine production technologies [13].
3. *Gavi, the Vaccine Alliance:* Founded in 2000, Gavi has supported governments in 73 countries to introduce and expand coverage of high-priority childhood vaccines, thereby reducing the volatility of the EPI vaccine market and stabilizing demand forecasts [7]. As highlighted in Fig. 1, Gavi procurement (through UNICEF) has more than tripled in the past ten years to more than $1.7 billion annually, or about 4% of the global vaccine market value and about 2.8 billion doses [14], [15]. In addition, UNICEF and its partners continue to improve procurement strategies, offering long-term contracts that enable them to negotiate lower prices earlier in a vaccine’s product cycle [16].

**Fig. 1:**
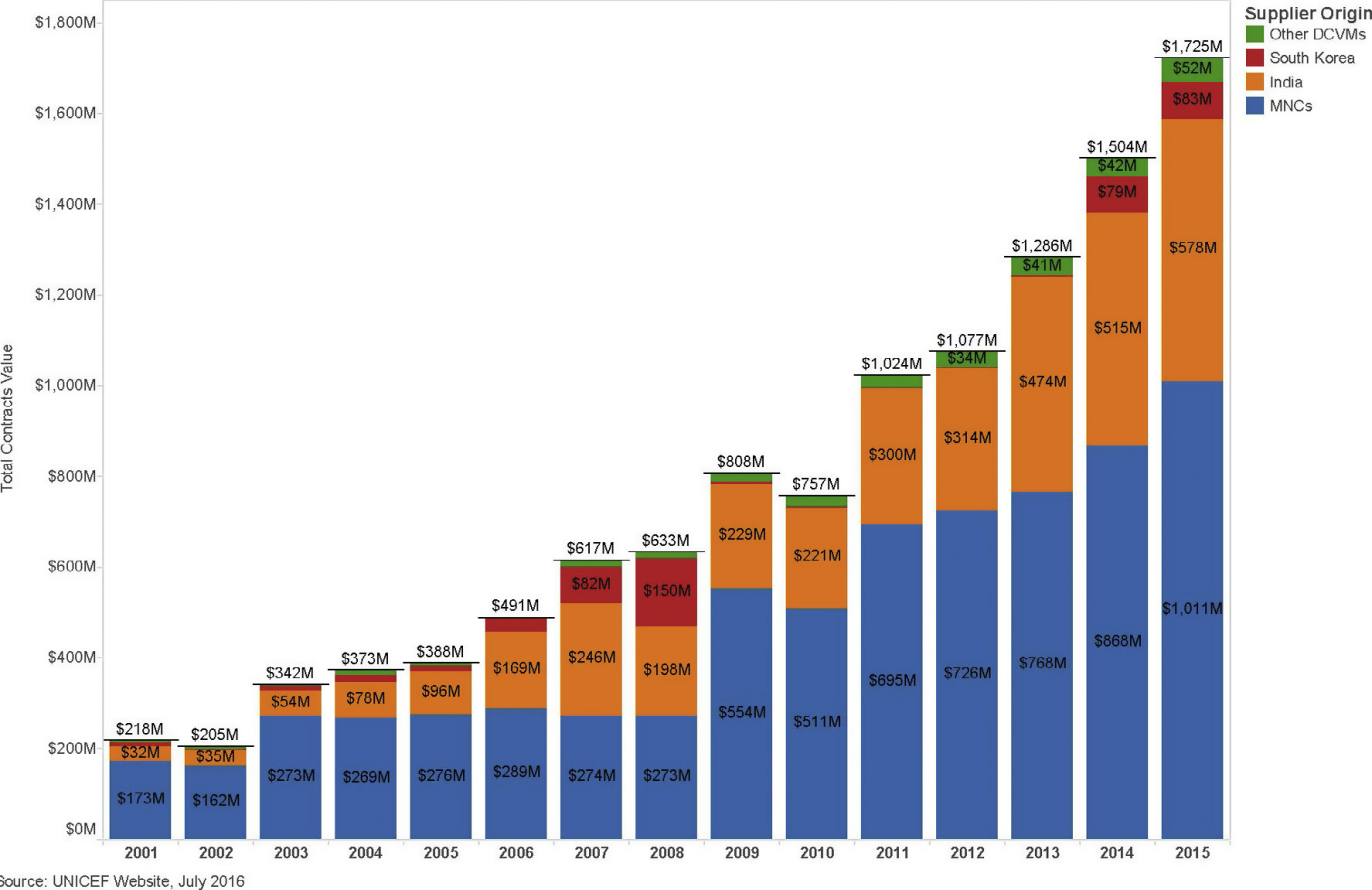
Supplier origins and values of UNICEF vaccine purchases over time. *Abbreviations used in the figure:* DCVM: developing-country vaccine manufacturer; MNC: multinational corporation.
  Selling through UNICEF requires WHO prequalification, an intensive process to ensure vaccines meet global standards of quality, safety, and efficacy [17]. Achieving prequalification requires long-term commitment on the part of the firm and the host government (in development of an NRA), and only a limited number of DCVMs have been able to prequalify their vaccines.
4. *Increased competition and participation of DCVMs:* As Gavi purchasing increases total global market volume, pricing for some vaccines has become more competitive, and DCVMs are vying for, and winning, market share. In 2001, only one of the five manufacturers that supplied vaccines to Gavi was based in a developing country. By 2015, more than half of vaccine suppliers were based in developing-country markets [18]. DCVMs produced 41% of all UNICEF-procured vaccines (in value terms) in 2015, an increase from roughly 20% in 2001 [9], [19]. Pentavalent vaccines provide a good example, growing from one, multinational corporation (MNC) supplier in 2001 to six, Indian and South Korean producers in the procurement period for 2017–2019, and with the average weighted price declining by 76%, to less than one US dollar [20], [21].

### 3.2 Vaccine production viability framework

Table 2 lists the original 1997 viability factors and includes modifications to the sub-elements to reflect the current environment. We also added one additional factor, the “enabling environment,” and review all factors below.

**Table 2:** 2016 updated viability factors from Milstien et al. [1]. Changes in bold denote added factor or sub-element.

Viability factor	2016
Economies of scale and volume; product portfolio	• Number of vaccines manufactured >2 • **Depending on the technology – production volumes on par with global average**

cGMP and consistency of production	• Percentage of lots failed <5% • Consistent number of lots per year • Consistent number of doses per lot • Maintenance program and budget • Planned, significant capital expenditure per year • Quality assurance budget and program • **WHO-prequalified product(s) or plan to reach prequalification**^b^

Functioning NRA—assurance of quality	• Customer has choice • NRA with six functions^a^: 1. Published set of requirements for licensing 2. Surveillance of vaccine field performance 3. System of lot release 4. Use of laboratory when needed 5. Regular inspections for cGMP 6. Evaluation of clinical performance • NRA is an independent authority

Systems to access new technologies and support research and development	• Process development budget and program • Research budget and program • **Realistic plan to meet defined market needs (national, international, or combination)** • **Added new technology in last five years or joint ventures, collaborations, or other technology transfers in last five years** • **Able to access and license intellectual property**

Historical performance	• **Supply sufficient to meet target market demand** • Proven scale-up in last five years

**Government policies and enabling environment for investment and regulation**	• **Government supporting WHO recognition of NRA** • **Government incentives for production of vaccines** • **Government supports partnerships with international research organizations and manufacturers**

Management structure	• **Detailed three-year strategic plan** • Human resources training plan (critical for cGMP and prequalification) • Appropriate ratio of skilled/unskilled staff • **Ability to access capital sources**

Legal status/autonomy	• Control to set salaries • Control to hire and fire as necessary • Control over revenues, budgets • **Price covers full cost per dose** • Political stability


cGMP: current Good Manufacturing Practice; NRA: national regulatory authority (formerly national control authority); WHO: World Health Organization.

a See the WHO immunization standards website for more detail: http://www.who.int/immunization_standards/national_regulatory_authorities/role/en/.

b WHO prequalification will be necessary for manufacturers requiring access to international markets to achieve economies of scale; however, some manufacturers, operating in very large markets, with protections against competition, may be able to reach scale in which WHO prequalification is not necessary.

#### 3.2.1 Economies of scale and volume; product portfolio

Vaccine production has high fixed costs, and the industry relies on economies of scale to distribute these costs across millions of doses, thus lowering final prices. At a minimum, to remain viable, public and private manufacturers need to price their vaccines to recover their full cost. Fixed costs (excluding labor), which are often 25–35% of total production costs, range from tens to hundreds of millions of dollars (depending on the technology and size of the facility), and distributing this cost across hundreds of millions of doses is critical for competitive pricing [22], [23], [24]. Furthermore, a broad product portfolio contributes to scale, allowing manufacturers to share the costs of facilities, management, and quality assurance across multiple products.

Milstien at al. hypothesized that either a minimum domestic population 50 million, indicating a birth cohort of approximately 1–2 million, or equivalent sized exports, was required to sustain a supplier [1]. Our research indicates that a much larger market size is needed to achieve economies of scale today. This can be achieved through (1) serving a large domestic market; (2) selling to large, global procurers (Gavi/UNICEF and PAHO), which agglomerates demand from customers; and/or (3) accessing regional markets through cross-border trade. Selling outside of national borders typically requires an established NRA and often WHO prequalification [25]. Experts indicated that meeting national demand is often the first step for vaccine manufacturers and is typically mandated for those receiving government support. Governments also often expect national manufacturers to match or undersell Gavi/UNICEF prices, creating pressure for firms to cut costs, potentially through high-volume production.

#### 3.2.2 Current Good Manufacturing Practice and consistency of production

Experts interviewed stressed the importance of quality and cGMP compliance as a critical factor for viability of all manufacturers, public and private alike [26], [27].^3^ Over the last 20 years, worldwide cGMP standards have become increasingly stringent. The requirements in both international and domestic markets for manufacturers to adapt and comply are both costly and require a change in management philosophy and in employee compliance. Examples of increased quality standards include increased documentation, data integrity, and facility and process validation [28]. Standards of regulations have targeted the quality of water purity used for solvents and washing, steam sterilization of lyophilizers, air filters for hot air sterilization of containers, clean rooms, and autoclaves [29]. To meet these requirements, more manufacturers are hiring experts with years of experience, often gained with MNCs, and taking advantage of WHO training and internationally accepted standards to ensure staff have appropriate expertise. [30], [31], [32].

#### 3.2.3 Functioning national regulatory authority—assurance of quality

Milstien et al. highlighted that a functioning NRA plays an essential role in ensuring the credibility of the quality of vaccines [1]. NRAs must be able to enforce quality requirements through licensing, surveillance of field performance, lot-by-lot scrutiny, laboratory testing, cGMP inspection of manufacturers, and evaluation of clinical trials [30]. Furthermore, for an NRA to provide credible, impartial oversight, it must be independent from corporate or political interference. While a manufacturer is responsible for cGMP production to produce high-quality vaccines, the NRA plays an essential government role in ensuring that quality is maintained, which may include closing facilities that do not meet standards [3], [33], [34].

WHO certification of an NRA’s ability to perform the six basic functions outlined in Table 2 has become a prerequisite for vaccine manufacturers aspiring to supply the international market. UNICEF, Gavi, and some countries rely on WHO-prequalified vaccines, produced in countries with WHO-recognized NRAs [25]. Substantial government commitment and investment are needed to achieve WHO certification; currently, only 14 developing^4^ and 23 developed^5^ countries have achieved certification for their NRA [35], [36].

#### 3.2.4 Systems to access new technologies and support research and development

As countries include combination and other complex vaccines in EPI schedules, manufacturers must continually innovate and invest in new technologies and production processes. This requires an explicit strategy and long-term planning. Technology transfer is an efficient mechanism leveraged by many DCVMs to advance product and technology development. Technology transfer typically follows one of two approaches. In the first, a public company or research institution develops a technology and transfers it to other manufacturers. This has been the case with polio vaccines, and more recently with Hib conjugate and meningococcal conjugate A and C vaccines [37], [38], [39]. In the case of Hib, Intravacc, a Dutch public research institution, transferred Hib conjugate vaccine technology to DCVM producers with large production capacity and a strong track record of high-quality vaccine production [29].While this method of transfer has proven to be successful when backed with sufficient financial and technical resources, declining budgets for publicly funded vaccine research institutes will likely reduce these opportunities [38].

The second route of technology transfer is a stepwise transfer from a corporate partner to a DCVM through a joint venture or technology transfer agreement. TRIPS limits more informal approaches to adopting technology [40]. As a result, companies relying on this approach typically proceed in phases, allowing partners to strengthen capacity in a reverse engineering of the process. The initial engagement may begin with labeling, move to fill and finish, and end with independent bulk vaccine production [13], [41], [42].

Technology transfer is a lengthy and expensive process that requires deep commitment as well as aligned value for each partner. Transfers from both public institutes and corporations take a minimum of four years for a vaccine to be consistently produced and available on the market [37], [41]. The transfers typically include significant investment in human resources to support high quality standards, making it an attractive vehicle for DCVMs. As a result, we updated the “New technology” viability factor (see Table 2) to include a realistic plan to meet market needs and the ability to access new technologies and license intellectual property.

#### 3.2.5 Historical performance

The nature of the vaccine market in which public and private DCVMs compete makes historical performance and reputation especially critical. Long lead times, multi-year supply contracts, and risk of supply/quality inconsistency make the cost of even small missteps particularly high, and procurers may be reticent to offer significant contracts to newcomers. Reputation may also be a strong factor in an MNC’s willingness to engage in technology transfer. The ability to consistently maintain a high-quality standard of vaccines in the home market is often a first step toward proving viability, while the ability to adapt and enter the market quickly with new products can lead to growth.

#### 3.2.6 Government policies and enabling environment for investment and regulation

Government commitment, policies supporting access to capital, and continuous sponsorship of an independent NRA are critical for the long-term viability of manufacturers. The growth of vaccine manufacturers in many developing countries is linked to the support the manufacturers receive from their governments. While overall economic development has helped to increase public and private access to capital, the need for continued capital investment to ensure successful compliance with cGMPs and adoption of new production technology was noted as a particular challenge for manufacturers and important for governments to take into consideration. Furthermore, all interview respondents discussed the significant role of government support, including support for a WHO-certified, functional NRA that is appropriately positioned to engage with manufacturers. Many country governments have established policies to support the growth of the biotechnology and pharmaceutical industries, helped to enable collaborations with multi-national corporations, and committed to strengthening national immunization programs with secured funding to procure vaccines to grow and stabilize domestic demand [13], [34], [42].

#### 3.2.7 Management structure and legal status/autonomy

A management structure that allows a manufacturer to make staffing and investment decisions and strategic and operational plans remains essential for viability. Interviewees highlighted that a critical factor in assessing a DCVM for a technology transfer or other supply agreement is the organization’s culture and management structure—effectively, the ability and commitment of its leadership to take the necessary steps to ensure affordable, high-quality supply. As highlighted by Milstien et al., parastatals and public entities can compete as long as they have an appropriate level of autonomy; without management autonomy, political interference in the company’s operations can undermine its viability [1].

### 3.3 Inferences about manufacturer viability

We were able to identify and subsequently analyze 27 of the 31 firms included in the 1997 study. Table 3 shows the 1997 status of all 31 manufacturers and provides the current viability ratings of the 27 included in this study.^6^ Only four of the original manufacturers failed. Another two were reorganized. One firm considered ‘high probability’ was fully restructured into a public-private partnership, while the others survived, indicating the manufacturers were able to sustain production and adapt to changes. While 23 of the 27 manufacturers studied continue to produce vaccines, several have experienced significant challenges.

**Table 3:** Current status of developing-country vaccine manufacturers reviewed in 1997 [43], [44], [45], [46], [47].

Number of firms…	High probability of viability	Potentially viable	Low probability of viability	Total number analyzed
In original paper	5	14	12	31
Identified and reviewed	5	14	8	27
No longer operating		1	3	4
Surviving in 2016	5	13	5	23

Table 4 presents selected viability data for the 23 of 27 manufacturers that currently produce vaccines. The data were selected because they are measurable and publicly available. Four of the five “low-probability” entities underwent significant reorganization and experienced significant production issues. Seven of the 12 “potentially viable” firms have also had production challenges. A large majority of the entities, 21 of 23, currently operate in a country with an NRA certified as functional by WHO, and 15 of 23 have cGMP facilities, an indication of quality and consistency. Only eight of the reviewed manufacturers achieved WHO prequalification for their vaccines. Even though some manufacturers may aim to produce only for a national market, this remains a stark indicator that DCVMs that started as government owned and operated continue to struggle to achieve WHO prequalification to compete in international markets.

**Table 4:** Developing-country vaccine manufacturers reviewed and select measurable characteristics reflecting viability metrics [43], [44], [45], [46], [47].

Firm characteristics	High probability of viability (N = 5)	Potentially viable (N = 13)	Low probability of viability (N = 5)	Relevant viability factors
Significant reorganization	3	6	3	• Management structure • Legal status/autonomy
Significant production issues	0	7	4	• Consistency of production • Enabling environment • Historical performance
Operating with cGMP facilities	5	8	2	• Consistency of production
Currently operating in a country with a functioning NRA	4	13	4	• Credibility of quality • Enabling environment
Currently producing WHO-prequalified vaccines	2	6	0	• Consistency of production • Credibility of quality

cGMP: current Good Manufacturing Practice; NRA: national regulatory authority; WHO: World Health Organization.

## 4 Conclusion

Changes in the market over the past 20 years shifted the playing field for DCVMs that were state-owned in 1997. Some successfully adapted, with governments shifting from a leading role in manufacturing to assuring quality through functioning NRAs and creating an enabling environment for vaccine manufacturers. Allowing manufacturers the autonomy to make timely, appropriate decisions is a strong indicator of success. Some did not adapt, with governments failing to create the enabling environment outlined in this paper placing their production and consumers at risk.

Some manufacturers were able to execute effective market strategies, hire talent, and engage with outside organizations to develop and acquire technology. In turn, this has allowed these manufacturers to more effectively respond to the changes in the market landscape and reliably meet demand with high-quality, affordable vaccines. To remain viable, manufacturers, whether public or private, will need to sustain robust strategies to stay abreast or ahead of the evolving market. Other manufacturers were shut down by their NRA, exited the market, or continue to struggle to produce vaccines, due to the absence of one or more of the critical viability factors.

Lastly, the rapidly changing technological environment and decrease in public funding for basic research has altered how manufacturers gain access to technology. Increasingly stringent rules around intellectual property, including TRIPS, have pushed many manufacturers toward joint ventures or technology transfers with MNCs or academic institutions. The success of these agreements will become increasingly important as new manufacturing techniques, multivalent vaccines, and more challenging cGMP standards are adopted at an increasingly rapid pace.

The viability framework outlined in 1997 was useful in identifying the strengths and weaknesses of DCVM manufacturers, most of which were publicly owned at the time. Our research indicates that updating the viability framework will ensure it continues to be a useful tool for governments, donors, and investors that are weighing the value of establishing or investing in a manufacturer. The updated viability framework provides insights into the investments, structure, size, policies, and management leadership that have, over two decades, proven essential to a sustained, affordable, high-quality supply of vaccines to meet national and global needs.

## Funding

This work was supported by the Bill & Melinda Gates Foundation, Seattle, WA [Grant No.: OPP1131997].

## Role of the funding source

The funder of this study had staff (co-authors of this manuscript) who had a role in study design, data analysis, data interpretation, or writing of the report. The corresponding author had full access to all data in the study and had final responsibility for all content and for the decision to submit for publication. The views presented in this article are solely those of the authors and do not represent the views of the Bill & Melinda Gates Foundation.

## Conflict of interest

None.

## Sources for data in the figure

1. United Nations Children’s Fund. Historical Vaccine Procurement, http://www.unicef.org/supply/index_38554.html; updated August 18, 2016 [accessed 03.11.16].
2. United Nations Children’s Fund. DTP-HepB-Hib vaccine supplier and pricing data 2001–2019 [table], http://www.unicef.org/supply/files/DTP-HepB-Hib.pdf; updated October 18, 2016 [accessed 03.11.16]^.^
3. United Nations Children’s Fund (UNICEF). Supply Annual Report 2015. Nordhavn, Denmark: UNICEF Supply Division; 2015.

## Footnotes

3 cGMPs are an evolving set of standards for manufacturers and are needed to ensure the production of safe, high-quality vaccines. cGMPs address the quality of facilities, inputs, control test procedures, production processes, and product specifications.

4 “Developing country” is defined as any country categorized as low or middle income by the World Bank. Those that have achieved WHO NRA certification are: Brazil, Bulgaria, China, Cuba, Egypt, India, Indonesia, Iran, Macedonia, Mexico, Russia, Senegal, Thailand, and Vietnam.

5 “Developed country” is defined as any country categorized as high income by the World Bank. High-income countries that have achieved WHO certification for their NRA are: Australia, Austria, Belgium, Canada, Croatia, Czech Republic, Denmark, Finland, France, Germany, Greece, Israel, Italy, Hungary, Japan, Netherlands, Poland, South Korea, Spain, Sweden, Switzerland, United Kingdom, and United States.

6 In cases in which limited data were available, we made inferences about the original categorization. The conclusions we draw are therefore intended to be directional, rather than absolute.

## References

[1] Milstien J., Batson A., Meaney W. (1997). A systematic method for evaluating the potential viability of local vaccine producers. Vaccine.

[2] Milstien JB, Batson A, Wetheimer AI. Vaccines and drugs: characteristics of their use to meet public health goals. World Bank: Health, Nutrition and Population (HNP) Discussion Paper. March 2005, <http://siteresources.worldbank.org/HEALTHNUTRITIONANDPOPULATION/Resources/281627-1095698140167/MilstienVaccinesDrugsFinal.pdf> [accessed 14.2.17].

[3] Danzon PM, Pereira NS. Vaccine supply: effects of regulation and competition. National Bureau of Economic Research (NBER) Working Paper 17205. Cambridge, MA: NBER; 2011.

[4] Ozawa S., Stack M.L., Bishai D.M., Mirelman A., Friberg I.K., Niessen L. (2011). During the ‘Decade of Vaccines’, the lives of 6.4 million children valued at $231 billion could be saved. Health Aff.

[5] Whitney C.G., Zhou F., Singleton J., Schuchat A. (2014). Benefits from immunization during the Vaccines for Children program era – United States, 1994–2013. MMWR.

[6] Ozawa S., Clark S., Portnoy A., Grewal S., Brenzel L., Walker D.G. (2016). Return on investment from childhood immunization in low- and middle-income countries, 2011–20. Health Aff.

[7] Gavi, the Vaccine Alliance. Working together for healthy vaccine markets, <http://www.gavi.org/about/gavis-business-model/making-vaccines-affordable/> [accesssed 23.06.16].

[8] World Health Organization (WHO), United Nations Children’s Fund (UNICEF). State of the World’s Vaccines and Immunization. Geneva: WHO; 1996.

[9] Kaddar M. (2013). Global vaccine market features [PowerPoint presentation].

[10] Gandhi G., Nguyen A. (2016). Changing market needs: vaccine markets today and beyond.

[11] US Centers for Disease Control and Prevention. Recommended Immunization Schedules for Persons Aged 0 through 18 Years United States, 2016, <http://www.cdc.gov/vaccines/schedules/hcp/imz/child-adolescent.html> [accessed 28.10.16].

[12] World Health Organization (WHO). Table 1. Summary of WHO position papers – recommendations for routine immunization, <http://www.who.int/immunization/policy/Immunization_routine_table1.pdf?ua=1>; September 2016 [accessed 28.10.16].

[13] Milstien J.B., Gaulé P., Kaddar M. (2007). Access to vaccine technologies in developing countries: Brazil and India. Vaccine.

[14] Kaddar M., Milstien J., Schmitt S. (2014). Impact of BRICS’ investment in vaccine development on the global vaccine market. Bull World Health Organ.

[15] United Nations Children’s Fund. Total number of doses (millions doses) and total value (millions USD) of each vaccine bought by year for 1996-2014; 2015, <http://www.unicef.org/supply/files/Total_vaccine_doses_procured_1996-2014.pdf> [accessed 28.10.16].

[16] Gavi, the Vaccine Alliance. Gavi, The Vaccine Alliance: Supply and Procurement Strategy 2016-20. Geneva: Gavi, the Vaccine Alliance; 2016.

[17] World Health Organization. Prequalification, <http://www.who.int/topics/prequalification/en/> [accessed 28.10.16].

[18] Gavi, the Vaccine Alliance. Developing country pharmaceutical industry, <http://www.gavi.org/about/partners/developing-country-vaccine-industry/> [accessed 28.10.16].

[19] United Nations Children’s Fund. Supplies and logistics: historical vaccine procurement, <http://www.unicef.org/supply/index_38554.html>; updated August 18, 2016 [accessed 28.10.16].

[20] United Nations Children’s Fund. Supply of children’s five-in-one vaccine secured at lowest-ever price [press release], <http://www.unicef.org/media/media_92936.html>; October 19, 2016 [accessed 28.10.16].

[21] United Nations Children’s Fund. DTP-HepB-Hib vaccine supplier and pricing data 2001–2019 [table], <http://www.unicef.org/supply/files/DTP-HepB-Hib.pdf>; updated October 18, 2016 [accessed 03.11.16].

[22] United Nations Children’s Fund (UNICEF). Summary of UNICEF study: a commercial perspective of vaccine study. New York: UNICEF; 1994.

[23] Datla M. Understanding vaccine manufacturing. Presented at: GAVI Alliance Partners’ Forum, December 5–7, 2012; Dar es Salaam.

[24] Mahoney R.T., Francis D.P., Frazatti-Gallina N.M., Precioso A.R., Raw I., Watler P., Whitehead P., Whitehead S.S. (2012). Cost of production of live attenuated dengue vaccines: case study of Instituto Butantan, Sao Paulo. Brazil. Vaccine.

[25] Dellepiane N., Wood D. (2015). Twenty-five years of the WHO vaccines prequalification programme (1987–2012): lessons learned and future perspectives. Vaccine.

[26] Topal C. A milestone for China and global health: implications of China’s first WHO-prequalified vaccine [interview with Jiankang (Jack) Zhang]. The National Bureau of Asian Research, <http://nbr.org/research/activity.aspx?id=375>; November 25, 2013 [accessed 24.03.16].

[27] Topal C, Luthra K. A pivotal moment for China and vaccine manufacturing [interview with Jiankang (Jack) Zhang]. The National Bureau of Asian Research, <http://nbr.org/research/activity.aspx?id=141>; May 25, 2011 [accessed 24.03.16].

[28] Dellepiane N., Griffiths E., Milstien J.B. (2000). New challenges in assuring vaccine quality. Bull World Health Organ.

[29] Milstien J., Costa A., Jadhav S., Dhere R. (2009). Reaching international GMP standards for vaccine production: challenges for developing countries. Expert Rev Vaccin.

[30] Hamidi A., Boog C., Jadhav S., Kreeftenberg H. (2014). Lessons learned during the development and transfer of technology related to a new Hib conjugate vaccine to emerging vaccine manufacturers. Vaccine.

[31] Milstien J., Dellepiane N., Lambert S., Belgharbi L., Rolls C., Knezevic I. (2002). Vaccine quality—can a single standard be defined?. Vaccine.

[32] Ward A. China’s biotech revolution ushered in by entrepreneurs. Financial Times, <https://www.ft.com/content/e916bf04-d6e2-11e5-829b-8564e7528e54>; March 7, 2016 [accessed 28.10.16].

[33] The Times of India. Pasteur Institute to start supplying DPT vaccines soon, <http://timesofindia.indiatimes.com/city/coimbatore/Pasteur-Institute-to-start-supplying-DPT-vaccines-soon/articleshow/30367602.cms>; February 14, 2014 [accessed 02.08.16].

[34] Reopening of closed vaccine units [press release]. Press Information Bureau, Government of India, Ministry of Health and Family Welfare, <http://pib.nic.in/newsite/PrintRelease.aspx?relid=81773>; March 27, 2012 [accessed 02.08.16].

[35] World Health Organization. National regulatory authorities (NRAs) and national control laboratories (NCLs) in countries producing vaccines prequalified for purchase by UN agencies, <http://www.who.int/immunization_standards/national_regulatory_authorities/offices/en/> [accessed 24.03.16].

[36] The World Bank. World Bank Country and Lending Groups, <https://datahelpdesk.worldbank.org/knowledgebase/articles/906519>; September 2016 [accessed 28.10.16].

[37] Beurret M., Hamidi A., Kreeftenberg H. (2012). Development and technology transfer of Haemophilus influenzae type b conjugate vaccines for developing countries. Vaccine.

[38] Blume S.S. (2005). Lock in, the state and vaccine development: lessons from the history of the polio vaccines. Res Policy.

[39] LaForce M.F., Konde K., Viviani S., Préziosi M.-P. (2007). The meningitis vaccine project. Vaccine.

[40] Gong W., Friede M., Sparrow E. (2011). Increasing access to vaccines through technology transfer and local production.

[41] Baijot M. Technology transfer and vaccines: the GSK experience. Presented at: WHO Workshop on Technology Transfer for Local Manufacturing Capacity of Vaccines; November 30, 2010; Geneva.

[42] Ho P.L., Miyaji E.N., Oliveira M.L.S., de Oliveira Dias W, Kubrusly F.S., Tanizaki M.M. (2011). Economical value of vaccines for the developing countries—the case of Instituto Butantan, a public institution in Brazil. PLOS Neg Trop Dis.

[43] Milstien J.B., Gellin G., Kane di M., Fabio F., Homma A. (1996). Global DTP manufacturing capacity and capability Status report: January 1995. Vaccine.

[44] Children’s Vaccine Initiative (1994). Vaccine self-sufficiency: improving local production and quality control.

[45] Chang-Blanc D., Brewer K. (1999). Motivations for local vaccine production.

[46] Milstien J. (1999). Local vaccine production: issues of quality and viability.

[47] Children’s Vaccine Initiative (CVI). CVI Mission Reports: Bangladesh (1992), Brazil (1994), Egypt (1992), India (1993), Indonesia (1993), Iran (1993), Mexico (1994), Nigeria (1996), Pakistan (1993), South Africa (1993), Thailand (1996), and Philippines (1993). Geneva: World Health Organization/CVI.

